# Large Language Model Applications for Health Information Extraction in Oncology: Scoping Review

**DOI:** 10.2196/65984

**Published:** 2025-03-28

**Authors:** David Chen, Saif Addeen Alnassar, Kate Elizabeth Avison, Ryan S Huang, Srinivas Raman

**Affiliations:** 1 Temerty Faculty of Medicine, University of Toronto, Toronto, ON, Canada; 2 Department of Systems Design Engineering, University of Waterloo, Waterloo, ON, Canada; 3 Department of Radiation Oncology, BC Cancer Vancouver, 600 W 10th Ave, Vancouver, BC, V5Z 4E6, Canada, 1 416-946-4501

**Keywords:** artificial intelligence, chatbot, data extraction, AI, conversational agent, health information, oncology, scoping review, natural language processing, NLP, large language model, LLM, digital health, health technology, electronic health record

## Abstract

**Background:**

Natural language processing systems for data extraction from unstructured clinical text require expert-driven input for labeled annotations and model training. The natural language processing competency of large language models (LLM) can enable automated data extraction of important patient characteristics from electronic health records, which is useful for accelerating cancer clinical research and informing oncology care.

**Objective:**

This scoping review aims to map the current landscape, including definitions, frameworks, and future directions of LLMs applied to data extraction from clinical text in oncology.

**Methods:**

We queried Ovid MEDLINE for primary, peer-reviewed research studies published since 2000 on June 2, 2024, using oncology- and LLM-related keywords. This scoping review included studies that evaluated the performance of an LLM applied to data extraction from clinical text in oncology contexts. Study attributes and main outcomes were extracted to outline key trends of research in LLM-based data extraction.

**Results:**

The literature search yielded 24 studies for inclusion. The majority of studies assessed original and fine-tuned variants of the BERT LLM (n=18, 75%) followed by the Chat-GPT conversational LLM (n=6, 25%). LLMs for data extraction were commonly applied in pan-cancer clinical settings (n=11, 46%), followed by breast (n=4, 17%), and lung (n=4, 17%) cancer contexts, and were evaluated using multi-institution datasets (n=18, 75%). Comparing the studies published in 2022‐2024 versus 2019‐2021, both the total number of studies (18 vs 6) and the proportion of studies using prompt engineering increased (5/18, 28% vs 0/6, 0%), while the proportion using fine-tuning decreased (8/18, 44.4% vs 6/6, 100%). Advantages of LLMs included positive data extraction performance and reduced manual workload.

**Conclusions:**

LLMs applied to data extraction in oncology can serve as useful automated tools to reduce the administrative burden of reviewing patient health records and increase time for patient-facing care. Recent advances in prompt-engineering and fine-tuning methods, and multimodal data extraction present promising directions for future research. Further studies are needed to evaluate the performance of LLM-enabled data extraction in clinical domains beyond the training dataset and to assess the scope and integration of LLMs into real-world clinical environments.

## Introduction

The advent of electronic health records (EHR) has allowed clinicians to leverage their access to vast amounts of longitudinal, patient-level clinical text data that inform patient diagnoses, prognoses, and management [1]. However, the majority of useful clinical data are stored as unstructured free text that requires manual extraction into meaningful clinical features; therefore, clinicians spend more time on administrative work reviewing EHRs instead of practising patient-facing medicine [1]. To address this task of extracting key attributes from unstructured clinical text, natural language processing (NLP) methods have classically applied rule-based and machine-learning methods to identify important entities in text and categorize them based on categories of interest [2]. For instance, the extraction of cancer staging information from clinical text requires an NLP algorithm to recognize references to cancer staging in clinical texts and categorize these references according to defined cancer staging nomenclature, such as the TNM classification of malignant tumors system.

Rule-based classification relies on domain expert-designed rules, heuristics, ontologies, and pattern-matching techniques to extract information from text. In contrast, machine learning-based approaches use statistical models trained on large-scale labeled text data to automatically learn patterns and generalize these learned competencies in data extraction to unlabeled testing data. The emergence of deep learning models, a subfield of machine learning that focuses on artificial neural network models with multiple processing layers, has been particularly effective at modeling the hierarchical structure of natural language and demonstrated superior performance across diverse NLP tasks, including but not limited to data extraction [3].

One particularly promising deep learning architecture, known as the transformer model, has gained worldwide attention for its generative language competency and strong performance in question answering, sentence completion, and sentence classification tasks compared to other deep learning models [4]. Deep learning–based transformer models may require less time and fewer resources needed to manually annotate training datasets compared to classical machine learning models and can better address nuanced edge cases in data extraction that may not be explicitly accounted for in rule-based data approaches [5,6]. However, these models are often limited by their need for large-scale computational resources and training data [7,8].

Modern LLMs are commonly built using adaptations of the transformer architecture and trained on large corpora of text to enable human-like natural language competency. Due to their extensive training dataset, LLMs such as BERT and GPT may have zero-shot capabilities, meaning they can perform tasks without prior task-specific training [9]. Emerging research on fine-tuning LLMs with custom datasets and prompt engineering for conversational LLMs has yielded promising performance improvements for specialized NLP tasks compared to baseline LLMs.

Given the longitudinal nature of cancer care, the vast amount of clinical text associated with cancer patient EHRs necessitates the development of automated methods for data extraction from these clinical records into structured data, which is useful for review by oncologists. The broad natural language competency of LLMs encourages the design of specialized LLM applications for data extraction from unstructured clinical text, reducing the oncologists’ time and effort spent in manually reviewing patient EHRs to extract key information to inform their clinical decision-making.

The emergence of several recent pilot studies of LLM-enabled data extraction prompts the need for a scoping review to map the current landscape, including definitions, frameworks, and future directions for this novel tool in clinical data extraction. This review seeks to address this gap in the literature by characterizing primary research articles that evaluated an LLM tool applied to data extraction from unstructured clinical text into structured data.

## Methods

We queried OVID Medline on June 2, 2024, using oncology (“neoplasms,” “cancer,” “onco,” “tumor”) and generative LLM (“natural language processing,” “artificial intelligence,” “generative,” “large language model”) keywords in consultation with a librarian. Non-English articles, nonprimary research articles, articles published before 2000, and articles published in nonpeer-reviewed settings were excluded. The full search strategy is detailed in Multimedia Appendix 1. Following the deduplication of articles (n=10) using the Covidence review management tool, the literature search yielded 817 articles for manual screening.

We conducted abstract screening followed by full-text screening of articles in duplicate (KA and SA), including primary research articles that tested a large language model, were applied in oncology contexts, and evaluated the performance of data extraction from text. The articles that evaluated an NLP-based algorithm that did not assess an LLM, were secondary research articles, applied in only nononcology settings, and did not evaluate or report the performance of data extraction from the clinical text were excluded. Screening conflicts were resolved through consensus discussion with a third reviewer (DC).

We extracted key study attributes from the included full-text papers in duplicate (KA and SA), including clinical domain, LLM attributes (eg, model, use of fine-tuning, use of prompt engineering), the dataset used for training and testing, primary study outcomes, model training methodology, and model evaluation processes. The LLMs were coded as baseline if they were applied “out of the box” without additional fine-tuning. LLMs were coded as (1) fine-tuned LLMs: the study described training the baseline LLM on a custom dataset intended to yield improved data extraction performance compared to the baseline LLM alone; (2) zero-shot LLMs: they were applied “out-of-the-box” without additional prompt engineering, (3) prompt engineered LLMs: the study described adaptations to prompting procedures, such as one-shot or few-shot prompting, designed to yield improved data extraction performance compared to the baseline LLM alone. Data extraction conflicts were resolved through consensus discussion with a third reviewer (DC).

The synthesis of extracted data involved grouping studies based on similarities in the evaluated specific model, clinical domain applied, and shared themes of strengths and limitations, based on outcomes reported by the studies. The appraisal process involved the completion of a standardized data extraction form to systematically code in duplicate (KA and SA) which articles commented on which themes of strengths and limitations, and the discrepancies were resolved through discussion (DC and SR). The risk of bias was assessed using ROBINS-I (Version 2) in duplicate (KA and SA), with conflicts resolved through consensus discussion with a third reviewer (DC). Cohen *κ* score was used to assess inter-rater concordance. This scoping review followed the PRISMA-ScR reporting guideline.

## Results

**Figure 1.**
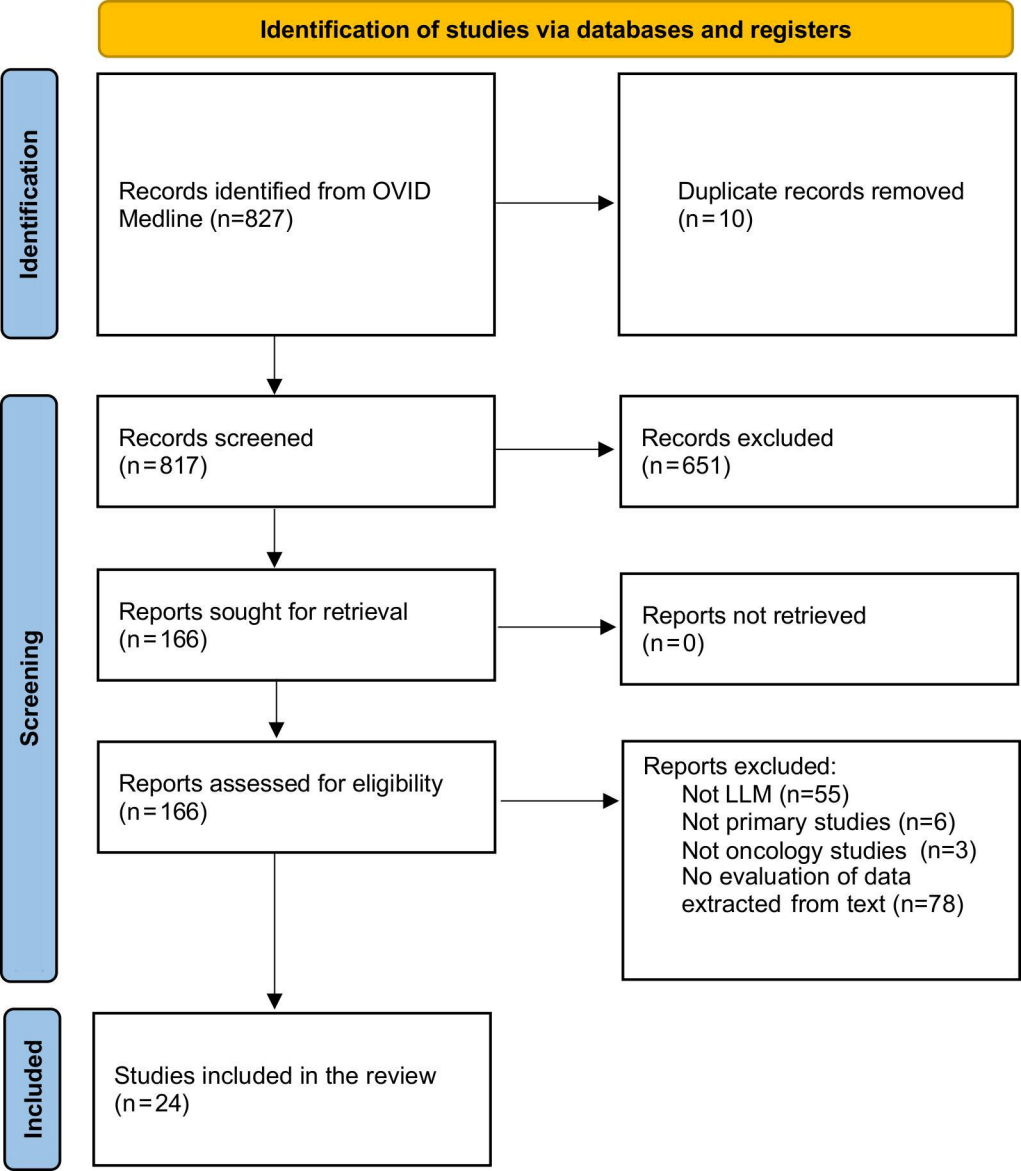
Search and filtering strategy used to select large language model studies evaluating data extraction performance for inclusion in this review. LLM: large language model.

**Figure 2.**
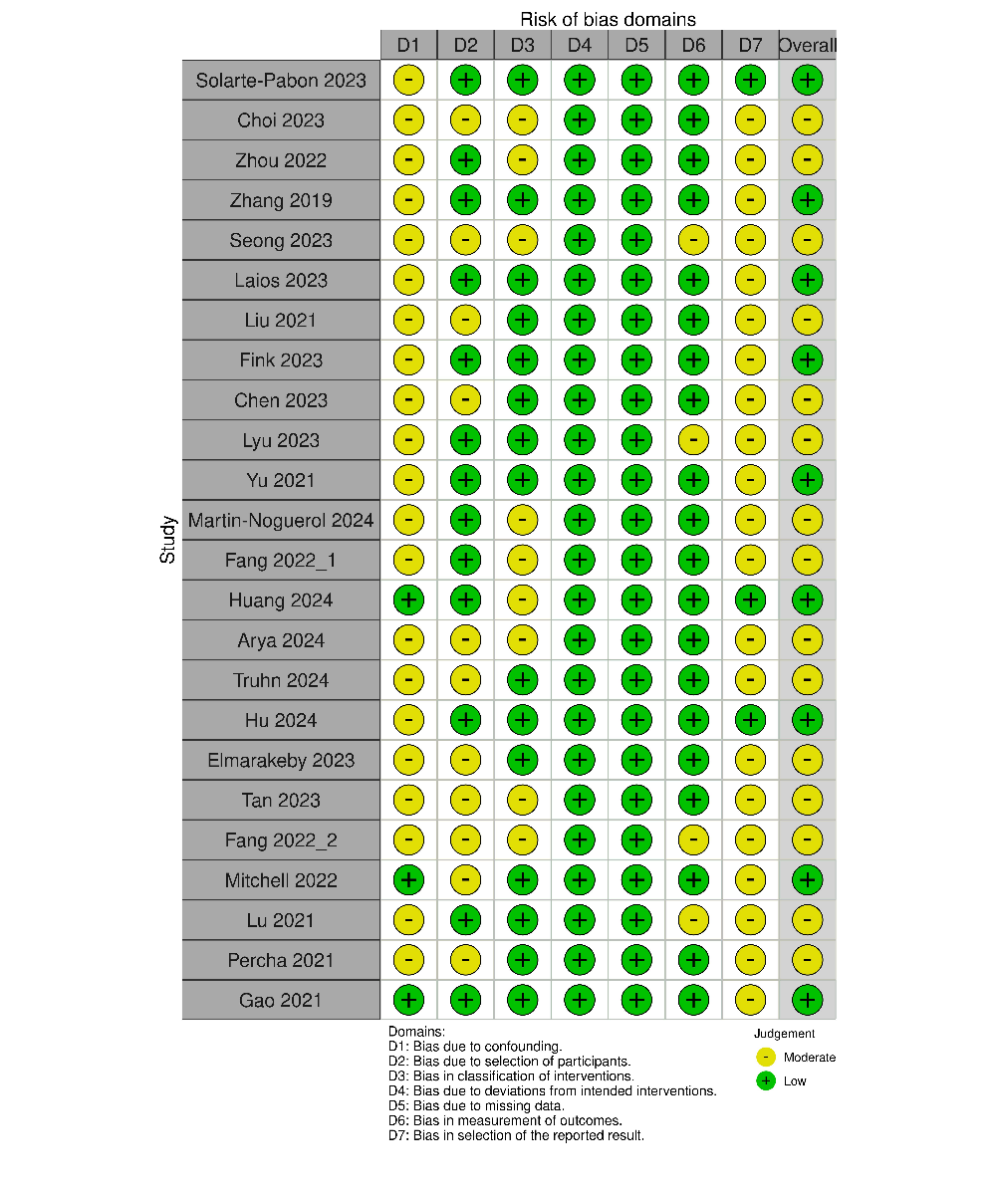
Risk of bias assessment using the ROBINS-I tool displayed as a traffic light plot for each included study [1,3,5-26].

The literature search yielded 817 papers, of which 24 papers met the inclusion criteria (Figure 1). Most included papers exhibited moderate (n=15, 62.5%) risk or low (n=9, 37.5%) risk of bias (Figure 2). The most common domains for moderate risk of bias included bias due to confounding (n=21, 87.5%) and bias in the selection of the reported result (n=21, 87.5%). No papers scored a high risk of bias in any domain. ROBINS-I risk of bias assessment exhibited moderate inter-rater concordance based on an *κ* score of 0.43.

Characteristics of the studies included in the study and published between 2019‐2024 are shown in Table 1. The most common LLMs reported in these studies included BERT and its variants, as well as ChatGPT. Additional details related to methodology are reported in Multimedia Appendix 2.

**Table 1.** Characteristics of studies included in the review.

Study ID	Clinical domain	Baseline model	Baseline or fine-tuned LLM^a^	Zero-shot or prompt -engineered LLM	LLM main outcomes
Solarte-Pabon 2023[10]	Breast	BERT; RoBERTa	Fine-tuned	Zero-shot	F-scores: BETA: 0.9371; Multilingual BERT: 0.9463; RoBERTa Biomedical: 0.9501; RoBERTa BNE: 0.9454
Choi 2023 [11]	Breast	ChatGPT-3.5	Baseline	Prompt-engineered	Accuracy: 87.7%
Zhou 2022 [3]	Breast	BERT	Fine-tuned	Zero-shot	F1-score: 0.866 and 0.904 for exact and permissive matches respectively
Zhang 2019 [1]	Breast	BERT	Fine-tuned	Zero-shot	NER:^b^ 93.53%; Relation extraction: 96.73% (best model, BERT+ Bi-LSTM-CRF)
Seong 2023 [5]	Colorectal	Bi-LSTM with a CRF layer; BioBERT	Fine-tuned	Zero-shot	Bi-LSTM-CRF:^c^ Precision: 0.9844; F1-score:0.9848; Pre trained word embedding performed better than the one hot encoding pre-processing
Laios 2023 [12]	Gynecology	RoBERTa	Baseline	Zero-shot	AUROC:^d^ 0.86; AUPRC:^e^ 0.87; F1: 0.77; Accuracy: 0.81
Liu 2021 [13]	Liver	BERT	Fine-tuned	Zero-shot	APHE^f^: 98.40%; PDPH^g^: 90.67%
Fink 2023 [14]	Lung	ChatGPT-3.5; ChatGPT-4.0	Baseline	Prompt-engineered	Overall accuracy: GPT-4: 98.6%; GPT-3.5: 84% Metastatic ID accuracy: GPT-4: 98.1%; GPT-3.5: 90.3% Oncologic progression accuracy: GPT-4 F1: 0.96; GPT-3.5: 0.91 Oncologic reasoning correctness: GPT-4: 4.3; GPT-3.5: 3.9 accuracy: GPT-4: 4.4; GPT-3.5: 3.3
Chen 2023 [15]	Lung	BERT	Fine-tuned	Zero-shot	Macro F1-score: Task 1:0.92; Task 2: 0.82; Task 3: 0.74
Lyu 2023 [16]	Lung	ChatGPT-4.0	Baseline	Zero-shot	Translate: 4.27/5; Provided specific suggestions based on findings in 37% of all cases
Yu 2021 [7]	Lung	BERT; RoBERTa	Fine-tuned	Zero-shot	BERT Lenient: 0.8999 BERT Strict: 0.8791
Martin-Noguerol 2024 [17]	Neurology	BERT	Fine-tuned	Zero-Shot	HGG: Precision: 79.17; Sensitivity: 76; F1:77.55; Metastasis: Precision: 73.91; Sensitivity: 77.27; F1: 75.56; AUC: 76.64
Fang 2022_1 [18]	Endocrine	BERT-BiLSTM-CRF	Fine-tuned	Zero-shot	Strict F1-score: 91.27%; Relaxed F1-score: 95.57%
Huang 2024 [19]	Pan-cancer	ChatGPT-3.5	Baseline	Prompt-engineered	Accuracy 0.89; F1 0.88; Kappa 0.80; Recall 0.89; Precision 0.89, Coverage 0.95
Arya 2024 [6]	Pan-cancer	BERT	Fine tuned	Zero-shot	Predict imaging scan site: Precision:99.4%; Recall:99.4%; F1-score: 99.3%; AUROC:99.4%; Accuracy:99.9%; Predict cancer presence: Precision:88.8%; Recall:89.2%; F1:88.8%; AUROC:97.6%; Accuracy:93.4%; Predict cancer status: Precision:85.6%; Recall:85.5%; F1-score: 85.5%; AUROC:97%; Accuracy:93.1%
Truhn 2024 [9]	Pan-cancer	ChatGPT-4.0	Baseline	Zero-shot	Experiment 1: Correct T-stage: 99%; Correct N-stage: 95; Correct M stage: 94; Lymph nodes; 99% Experiment 3: 100% accuracy
Hu 2024 [8]	Lung	ChatGPT-4.0	Baseline	Prompt-engineered	Prompt Base: Accuracy: 0.937; Precision: 0.860; Recall: 0.917; F1-score:0.882; Prior medical knowledge: Accuracy: 0.940; Precision:0.900; Recall: 0.864; F1:0.867; PMK-EN^h^: Accuracy: 0.896; Precision:0.871: Recall:0.776; F1: 0.786
Elmarakeby 2023 [20]	Pan-cancer	BERT	Fine-tuned	Zero-shot	AUC: ClinicalBERT: 0.93; DFCI-ImagingBERT: 0.95 F1: ClinicalBERT: 0.72; DFCI-ImagingBERT: 0.78
Tan 2023 [21]	Pan-cancer	GatorTron; BERT; PubMedGPT	Fine-tuned	Prompt-engineered	Accuracy: GatorTron: 0.8916; BioMegatron:0.8861; BioBERT:0.8861; RoBERTa:0.8813; PubMedGPT:0.8762; DeBERTa:0.8746; BioClinicalBERT: 0.8746; BERT: 0.8708
Fang 2022_2 [22]	Pan-cancer	BERT	Baseline	Zero-shot	ROC:^i^ 0.94
Mitchell 2022 [23]	Pan-cancer	BERT	Fine-tuned	Zero-shot	Group level site accuracy: 93.53%; Histology codes: 97.6%
Lu 2021 [24]	Pan-cancer	BERT	Fine-tuned	Zero-shot	Symptom domains: 0.931; problems with cognitive and social attributes on pain interference: 0.916; problems on fatigue: 0.929
Percha 2021 [25]	Breast	ALBERT; BART; ELECTRA; RoBERTa; XLNet	Fine-tuned	Zero-shot	ALBERT was the best-performing model in 22 out of the 43 fields
Gao 2021 [26]	Pan-cancer	BlueBERT	Fine-tuned	Zero-shot	BERT does not outperform baseline models–quantifiable measures not available

a LLM: large language model.

b NER: named entity recognition.

c Bi-LSTM-CRF: bidirectional-long short term memory-conditional random field.

d AUROC: area under the receiver operating characteristic.

e AUPRC: area under the precision-recall curve.

f APHE: hyperintense enhancement in the arterial phase.

g PDPH: hypointense in the portal and delayed phases.

h PMK-EN: Prior Medical Knowledge-English Prompt

i ROC: receiver operating characteristic.

**Figure 3.**
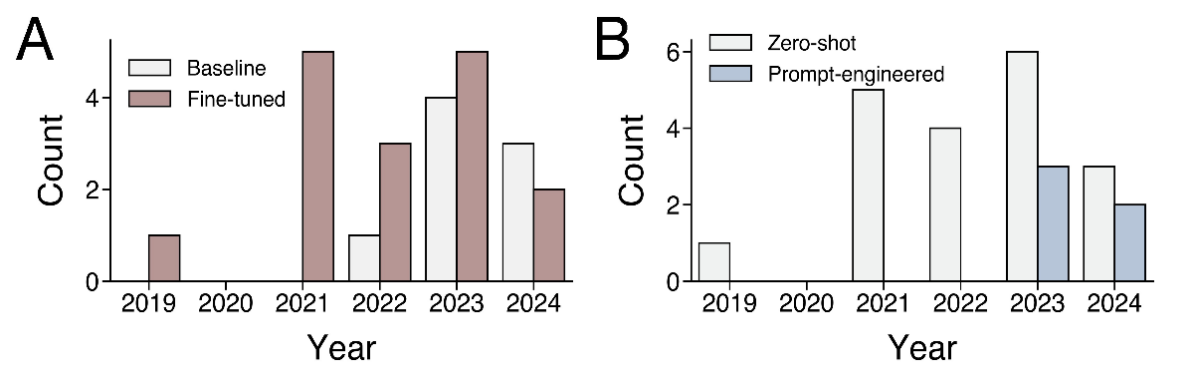
Number of studies that evaluated (A) fine-tuning and (B) prompt engineering methodologies to optimize large language model data extraction performance.

Most studies evaluated either the original or fine-tuned variants of the BERT LLM (n=18, 75%) in studies published between 2019‐2024, followed by the Chat-GPT conversational LLM (n=6, 25%), upon application to data extraction from clinical texts in oncology, in studies published between 2023‐2024. The LLMs for data extraction were commonly applied in pan-cancer clinical settings (n=11, 46%), followed by breast (n=4, 17%), lung (n=4, 17%), neurological (n=2, 8%), colorectal (n=1, 4%), gynecological (n=1, 4%), and liver (n=1, 4%) cancer contexts. The author teams of these studies belonged to institutions in the United States (n=11, 46%), China (n=4, 17%), Korea (n=3, 12%), Germany (n=2, 8%), Spain (n=2, 8%), the United Kingdom (n=1, 4%), and Singapore (n=1, 4%). Most studies were evaluated on datasets sourced from multiple institutional centers (n=18, 75%) compared to a single institutional center (n=6, 25%). Regarding the year of study publication, we observed a higher number of studies published between 2022‐2024 (n=18, 75%) compared to 2019‐2021 (n=6, 25%) (Figure 3). Notably, upon a comparison of studies published between 2022‐2024 with studies between 2019‐2021, the proportion of studies that reported the use of the fine-tuning method was lower (10/18, 55.6% vs 6/6, 100%) (Figure 3A), whereas the proportion of studies that reported the use of prompt engineering was higher (5/18, 28% vs 0/6, 0%) (Figure 3B).

## Discussion

### Principal Findings

Our scoping review of 24 studies highlights significant research interest in designing, evaluating, and deploying LLMs for data extraction from clinical text in oncology. The most commonly used LLMs for data extraction from clinical text in oncology include BERT and Chat-GPT, two of the most well-known LLMs in NLP research. These LLMs were most frequently applied in pan-cancer clinical contexts, reflecting their generalized natural language competency, regardless of clinical domain and context-specific terminologies and nomenclature. We observed a notable trend toward increasing utilization and refinement of LLM techniques over time, particularly in the areas of fine-tuning and prompt engineering. Given the common application of fine-tuning [26-28] and prompt-engineering [1,29,30] techniques in the design of deep learning- and LLM-based models in oncology, respectively, the emergence of optimized LLMs using these techniques represents a promising future direction for enhancing their data-processing capabilities. Despite these advancements, mixed reports of data extraction performance underscore the imperative for further assessment of these models across specific topics and use cases before their deployment as tools in cancer research and clinical care. Compared to historical statistical NLP and machine learning-based methods for data extraction in oncology, LLMs have been broadly evaluated for comparable applications, such as extracting tumor and cancer characteristics and patient-related demographic data [31].

The data processing competency of LLMs makes them a useful tool for automating repetitive, rule-based tasks, such as data extraction from clinical text on EHRs, to generate medical evidence about specific patients and patient populations that can inform patient care and population health guidelines respectively. Notably, LLMs have already shown competency in pilot studies of automated data extraction in biology [32], materials science [33], and pharmacology [33], suggesting their generalized ability to extract relevant named entities from the clinical text that may be useful to synthesize medical knowledge. Across the included studies in this review, we found that LLMs offer several benefits for data extraction in clinical oncology, though further benchmarking against representative datasets and classical machine learning or statistical NLP approaches is required to determine their superior performance. In general, LLMs exhibited positive performance metrics compared to baseline human or statistical NLP approaches or were deemed feasible and acceptable in cross-sectional studies. These LLMs harbor the potential to balance accuracy and efficiency when processing large-scale, complex, unstructured text datasets found in EHRs [19]. Using LLM approaches for clinical data extraction as a supportive tool along with a human reviewer may reduce the potential for errors associated with human-led manual data extraction alone, thereby enhancing the reliability of clinical data analyses and interpretations [34].

Moreover, LLMs may curtail the resources required for data extraction, which is traditionally a labor- and time-intensive process [35]. For instance, our review highlighted the generalized performance of LLM-enabled data extraction across various text types in oncology, including histological and pathological classification [9,36], imaging report classification [8,14], and data extraction from postoperative surgery reports [5]. By automating the extraction and preliminary analysis of clinical text data, these models may free up valuable time for health care professionals, allowing them to focus more on patient-facing care and synthesis of medical knowledge from LLM-extracted information rather than the burden of administrative data management [10,12,37]. This shift not only improves clinical efficiency and cost-effectiveness but also reduces the serious risks of burnout among clinical staff by mitigating some of the repetitive administrative tasks associated with data handling [11,38].

Additionally, the versatility of LLMs across different clinical text contexts is notable. Whether dealing with structured data formats or the myriad forms of unstructured data present in EHRs, such as physician’s notes and diagnostic reports, the general human-like natural language competencies of LLMs enable these “out-of-the-box” solutions to automatically adapt to and extract relevant information from varied data sources. This adaptability is crucial in precision oncology, where data from multiple data formats—such as imaging reports, next-generation sequencing results, and laboratory results—must be integrated and analyzed to generate personalized patient profiles and treatment strategies [39]. Our review highlighted that current state-of-the-art evaluations of LLMs for data extraction in oncology have primarily focused on clinical text as input. However, we also highlight the recent emergence of multimodal LLMs capable of processing both image- and text-based inputs, serving as a new frontier for clinical decision support [40]. Taken together, future research to optimize data extraction for specific text formats in oncology—each with their own nuances—may improve extraction accuracy, enhance reliability, and produce results that can be trusted by clinicians and readily inform clinical decision-making [41].

The distribution of studies included in our scoping review reflects a predominant application of LLMs in pan-cancer clinical domains, accounting for nearly half of all research studies. This suggests that researchers leverage the versatility of LLMs to address broad oncological challenges across multiple cancer types, likely due to the generalizable nature of these models for various cancer data [42]. Breast and lung cancer also constituted a large portion of the studies, which can likely be attributed to their high prevalence and extensive clinical data availability, providing a rich dataset for deploying and testing the efficacy of LLMs [43]. The focus on these specific cancers indicates a targeted approach, where models are fine-tuned to address unique data extraction challenges, such as cancer type-specific nomenclature and lexicons. This underscores the potential of LLMs to be customized for specialized medical fields while also highlighting their broad “out-of-the-box” utility in general oncology. For instance, Gao et al [44] reported that BlueBERT did not outperform baseline nonLLM models in pan-cancer contexts, while Fang et al [22] and Mitchell et al (2022) [23] reported that the data extraction performance of BERT exceeded 90% accuracy in pan-cancer contexts. The mixed performance reported by different pilot studies of data extraction performance within the same clinical domain may be confounded by study-specific factors, including the prompting methodology, benchmark dataset, and definitions of performance metrics. These findings align with similar reports of mixed performance across different tasks and clinical text datasets within cancer type-specific domains [45-47], highlighting the need for systematic benchmarks to assess LLM data extraction reliability and domain-specific limitations. Standardizing performance metrics and defining critical thresholds for acceptable performance of data extraction accuracy remain open research questions to be addressed.

Our analysis reveals an increasing trend in the use of fine-tuning and prompt-engineering techniques in studies on LLMs, with 16 (67%) studies incorporating fine-tuning and 5 (21%) using prompt engineering. This progression suggests a maturation in the application of LLMs in clinical settings, where research has transitioned from developing baseline models for simple data extraction to the optimization of existing models using novel model adaptations and prompting methodologies tailored to the intricacies of medical data extraction. Fine-tuning allows models to adapt to the unique linguistic and contextual challenges presented by medical texts, potentially improving the accuracy and relevance of extracted information [29]. In comparison, prompt engineering enables the creation of more effective queries that align closely with the specific information needs of specialty fields such as oncology, steering LLMs toward more precise data retrieval [48]. For instance, Huang et al [19] demonstrated that providing LLMs with example outputs for few-shot learning and chain-of-thought reasoning methods for prompting yielded higher classification performance compared to baseline zero-shot applications of LLMs for data extraction. The careful design of prompting methodologies personalized to specific tasks and clinical domains within oncology may yield more accurate and efficient data extraction performance [49].

Despite the promising applications of LLMs in clinical oncology, our review also highlights notable disadvantages, particularly in cases of poor data extraction accuracy and performance [8,9]. Among the 24 reviewed studies, 9 (38%) cited accuracy as a limitation of LLMs for data extraction. These shortcomings underscore the critical need for cautious integration of LLMs into clinical workflows. The variability in performance can be attributed to the complex and diverse nature of clinical data, which may include nuanced medical terminologies and varied presentation styles across different documents [50]. These challenges emphasize the necessity for ongoing refinement and testing of these models under real-world conditions. Another minor disadvantage is the token limit of many LLMs, including both BERT and ChatGPT [20,42,44]. This limitation may complicate the extraction process, requiring models to be adapted to longer texts and resulting in reduced performance of these models [51]. Future research directions, as indicated by the reviewed studies, should involve performance benchmarks against existing statistical and machine learning–based methods and the extension of LLM tool validation to external, hold-out cohorts from additional clinical domains beyond those used in initial training datasets [7,16,24]. This would help ensure that the models are robust and reliable across various medical specialties and global oncology patient populations. While LLMs hold significant potential to revolutionize data management in oncology, their integration into clinical practice must be approached with careful planning and systematic evaluation to truly harness their capabilities without compromising patient care quality and privacy. The interpretation of both advantages and disadvantages of LLMs requires individualized consideration of each study, on a case-by-case basis given the heterogeneity in benchmark datasets, study designs, and reported outcomes.

### Limitations

We acknowledge the limitations inherent in our scoping review. First, the rapid evolution of LLM technologies means that newer advancements may not have been fully represented in the reviewed studies due to the delays in publication cycles, leading to the omission of recent models. Second, the heterogeneity in study designs, datasets, and methodologies across included articles may affect the generalizability of findings in external contexts not evaluated in the same conditions as the original studies. Third, the majority of included studies originated from high-resource settings, primarily the United States, which may limit the applicability of results to lower-resource or structurally different health care systems. Fourth, while the risk of publication bias was not formally evaluated in our review, the tendency to publish studies with positive results may overrepresent the strengths of these LLMs without an understanding and consideration of their limitations and nonpublished, negative results. Fifth, more recent journals that publish artificial intelligence research may not be indexed in the search databases yet, limiting the completeness of the search results in this scoping review. Sixth, this scoping review searched only one literature database, which may have resulted in the omission of relevant studies from other sources and limited the comprehensiveness of the findings.

### Conclusion

In conclusion, the application of LLMs in oncology represents a forward leap in the digital transformation of health care data management. The potential to enhance data extraction processes and improve clinical decision-making is significant yet tempered by the current technological and methodological limitations. Ongoing research and development will be vital in harnessing the full potential of these models, ultimately leading to their more widespread adoption in clinical practice.

## Supplementary material

10.2196/65984 Multimedia Appendix 1 Scoping review full search strategy for MEDLINE.

10.2196/65984 Multimedia Appendix 2 Methodology characteristics of included studies.

10.2196/65984 Checklist 1 PRISMA-ScR reporting guideline.

## Abbreviations

EHR: electronic health record
LLM: large language model
NLP: natural language processing
