# Supplementary material for: Large Language Model Applications for Health Information Extraction in Oncology: Scoping Review
Source: JMIR Cancer. 2025 Mar 28;11:e65984. doi: 10.2196/65984 (PMC11970800; doi:10.2196/65984)
Supplement: Multimedia Appendix 1 [file cancer-v11-e65984-s001.docx]

| **Set** | **Search Statement** |
| --- | --- |
| 1 | exp Neoplasms/ |
| 2 | neoplas*.tw,kf. |
| 3 | cancer*.tw,kf. |
| 4 | onco*.tw,kf. |
| 5 | tumo?r*.tw,kf. |
| 6 | exp Natural Language Processing/ |
| 7 | (artificial* intelligen* adj5 (generat?? or generative)).tw,kf. |
| 8 | ((AI or AIs) adj5 (generat?? or generative)).tw,kf. |
| 9 | ((AI or AIs) adj5 chat*).tw,kf. |
| 10 | large language model*.tw,kf. |
| 11 | or/1-5 |
| 12 | or/6-10 |
| 13 | (exp animal/ not (exp animals/ and exp humans/)) or ((exp animals/ or exp animal experiment/) not humans/) or (in vitro or in vivo or cell lines).ti. |
| 14 | 11 and 12 |
| 15 | 14 not 13 |
| 16 | limit 15 to (clinical conference or clinical trial protocol or comment or consensus development conference or consensus development conference, nih or editorial or letter or news or newspaper article or preprint) |
| 17 | 15 not 16 |
| 18 | limit 17 to yr="2000 -Current" |
| 19 | limit 18 to english language |
